# The effect of immunological status, *in-vitro* treatment and culture time on expression of eleven candidate reference genes in bovine blood mononuclear cells

**DOI:** 10.1186/s12865-015-0099-7

**Published:** 2015-05-30

**Authors:** Mehdi Emam, Kathleen Thompson-Crispi, Bonnie Mallard

**Affiliations:** Department of Pathobiology, Ontario Veterinary College, University of Guelph, Guelph, ON Canada; Center for Genetic Improvement of Livestock, University of Guelph, Guelph, ON Canada

**Keywords:** Reference gene, Real time RT-PCR, Blood mononuclear cells

## Abstract

**Background:**

Technical feasibility of RNA quantification by real time RT-PCR has led to enormous utilization of this method. However, real time PCR results need to be normalized due to the high sensitivity of the method and also to eliminate technical variation. Normalization against a reference gene that is constitutively transcribed and has minimum variation among samples is the ideal method. Nevertheless, many studies have shown that there is no general reference gene(s) with ideal characteristics and candidate reference genes should be tested before being used as a “normalizer” in each study.

**Methods:**

The current study investigated the effects of previous exposure of the host to experimental test antigens and culturing time on the expression of 11 candidate genes when blood mononuclear cells (BMCs) were cultured and treated *in-vitro* by hen egg white lysozyme, *Candida albicans* extract and a mitogen. Mononuclear cells were isolated and cultured from 12 bovine blood samples representing 3 different immunological statuses. The expression of candidate housekeeping genes were measured by real-time RT-PCR at 4 and 24 hours post culture. The expression of candidate genes were first compared between the two time points in untreated samples. Constitutively expressed genes were further tested in linear mixed effects models to examine the effect of previous host exposure and *in-vitro* treatments.

**Results:**

Our findings showed that the expression of the most common reference genes, *β-actin,* and *Glyceraldehydes-3-phosphate dehydrogenase* (GAPDH), are significantly decreased at 24 hours after culturing BMCs, even without any treatment. The effect of culturing time was also significantly influenced the expression of *18s ribosomal RNA*, *β2-microglobulin*, *Tyrosine 3-monooxygenase/tryptophan 5-monoxygenase activation protein, zeta polypeptide* (YWHAZ) in BMCs. Only the expression of *C-terminal binding protein 1* (CTBP1) and *RAD50* among all tested genes were consistent after treatment of cultured BMCs with *C. albicans* whole yeast extract and Hen Egg White Lysozyme (HEWL), respectively. In addition, expressions of CTBP1, and RAD50 were independent from previous exposure of the host to the antigen.

**Conclusions:**

The results of this study demonstrated inconsistent expression of commonly used reference genes in untreated cultured BMCs over time. As this condition applies to negative controls in real time RT-PCR study designs, normalization against these genes can largely deceive the outcome, especially in kinetic studies. Moreover, the potential effects of immunological memory on the expression of reference genes should be considered if BMCs are collected from different individuals under different environmental conditions and if these cells are treated *in-vitro* by an antigen.

## Background

Quantification of mRNA expression to investigate the cellular mechanism in both physiological and pathological state of cells or tissues has been a method of choice in many research projects [1–3]. Various techniques including northern blotting, microarray, and real-time RT-PCR have been developed to help researchers to quantify very small amounts of mRNA, accurately [4]. Nowadays, the analysis of mRNA expression by real-time RT-PCR is the most feasible method due to the low cost, high sensitivity, and possibility of in-house species-specific designing of primers/probes. Despite of the advantages of this technique, the results, and procedure need to be verified due to the several pitfalls which may cause unreliable results [5]. A normalization step that removes variation in the quantity of starting material and efficiency of amplification between samples and runs, can greatly affect the reliability of quantitative RT-PCR results. Several methods have been proposed to normalize data such as normalization according to weight/volume of sample, quantity of RNA and/or using internal control genes [1, 3].

Housekeeping genes (HKGs) are considered to be expressed constantly and have been widely used as an internal control or reference gene. However, if the expression of the internal control genes are affected by the experimental procedure (i.e. treatments) then the results would not be valid [1]. Recently, expression stability of many HKGs have been investigated in different cells and tissues, including animals, and plants tissues, under treatment and/or untreated condition [1, 6–10]. The common conclusion from those studies emphasized that there is no general housekeeping gene to be used as a reference gene among all cells and tissue types, as well as under different treatments. On the other hand, in any experiment that aims to study gene expression, it is necessary that a gene or a set of genes that are not affected by the treatment or cell/tissue type are identified to be used as an experimental control [1].

The relative quantification of mRNA by real-time RT-PCR is a preferred method, particularly when species specific monoclonal antibody are lacking to analyze cellular phenomenon [11]. Nevertheless, quantification of mRNA in cultured cells, in particular those of the adaptive immune system, is complicated by several factors including immunological memory, *in-vitro* cell culture, and treatments by mitogens and/or antigens. Thus, many genes, including housekeeping genes, which may be constitutively expressed in non-immune system cells are likely differentially expressed in cells of the immune system. In the current study, the stability of the expression of eleven housekeeping genes (18s, 24s, ACTB, B2M, CTBP, GAPDH, MDM4, PPIA RAD50, SDHA, and YWHAZ) was analyzed in cultured bovine blood mononuclear cells (BMCs). BMCs were isolated from individuals with different *in-vivo* exposure to test antigens (immunological status) and treated *in-vitro* with a mitogen or a type 1 or type 2 test antigen. The hypothesis being that the history of host exposure to test antigens and culturing time affect the expression of housekeeping genes. The objective of this study was to identify potential instability of HKG expression in BMCs in immunological research due to immunological memory and experimental treatment when samples are collected from an outbred species in uncontrolled environmental settings.

## Results

### Amplification accuracy and efficiency

The melting curve analysis was carried out for each gene after the amplification step to evaluate the accuracy and uniformity of amplified product. One single peak for each gene was observed for all samples (data not shown) which represented an appropriate amplification. Quantification cycles (Cq) for each gene were determined using LightCycler® 480 software and then were analyzed by SASqPCR macro code [12]. The statistical results of the amplification performance are presented in Table 1. The amplification efficiency ranged from 0.79 to 0.98 for all primer pairs except 18s and Peptidylprolyl isomerase A (PPIA) which had the lowest efficiency, 0.57, and 0.70 respectively. The correlation coefficient (R^2^) was higher than 0.98 for all amplifications which represented the accurate estimation of efficiency. The lowest intercept was observed for *β*-actin (ACTB) and 18s with 19.8 and 25.3 respectively.

**Table 1:** Statistical assessment of the amplification performance of 11 candidate genes in bovine blood mononuclear cells

Gene*	**Slope**	**StdErr**_**s**_	**P value**_**s**_	**Intercept**	**StdErr**_**i**_	**P value**_**i**_	**R** ^**2**^	**E**
**18s**	−5.06151	0.0758	7.586E-10	25.3305	0.14632	2.507E-12	0.99866	0.57605
**24s**	−3.79032	0.05825	8.863E-10	31.739	0.11246	1.335E-13	0.99858	0.83582
**ACTB**	−3.368	0.064719	3.379E-09	19.8495	0.12108	3.475E-12	0.99779	0.98113
**B2M**	−3.93206	0.01988	1.127E-12	31.9222	0.03838	2.038E-16	0.99985	0.79606
**CTBP1**	−3.28926	0.09434	3.7E-08	35.1283	0.18211	1.31E-12	0.99509	0.98619
**GAPDH**	−3.46422	0.03558	7.917E-11	31.7187	0.0687	6.966E-15	0.99937	0.94386
**MDM4**	−3.73883	0.0703	3E-09	38.6855	0.13572	1.258E-13	0.99788	0.85124
**PPIA**	−4.31421	0.027696	4.722E-12	35.6981	0.058222	1.27E-15	0.99975	0.70527
**RAD50**	−3.94515	0.1124	4E-09	39.6633	0.20941	3.018E-14	0.99435	0.79257
**SDHA**	−3.69503	0.1683	1.03E-07	36.2313	0.30674	8.221E-13	0.98569	0.86481
**YWHAZ**	−3.55114	0.03416	5.343E-11	32.1879	0.06595	4.993E-15	0.99945	0.91249

Slope: Slope of standard curve; StdErr_s_: Standard error of the slope; P_value_s_: Statistic significance of the slope estimation; Intercept: Intercept of standard curve; StdErr_i_: Standard error of the intercept; P value_i_: Statistic significance of the intercept estimation; R^2^: the correlation coefficient; E: Efficiency of amplification. * 18s: 18S ribosomal RNA; 24s: Ribosomal protein S24; ACTB: Actin, beta; B2M: Beta-2-microglobulin; CTBP1: C-terminal binding protein 1; GAPDH: Glyceraldehydes-3-phosphate dehydrogenase; MDM4: mouse double minute 4 protein; PPIA: Peptidylprolyl isomerase A; RAD50: RAD50 homolog (S. cerevisiae); SDHA: Succinate dehydrogenase complex, subunit A; YWHAZ: Tyrosine 3-monooxygenase/tryptophan 5-monoxygenase activation protein, zeta polypeptide.

### mRNA expression of HKGs in cultured BMCs without treatment

#### The expression of candidate genes is affected by time in non-treated cultured BMCs

**Fig. 1:**
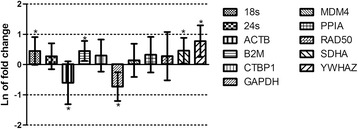
Mean and standard error of the natural log of RNA expression in untreated blood mononuclear cells at 24 h relative to 4 h of culture. * Shows the mean is significantly (*P* ≤ 0.05) different than zero based on T-distribution test (Forthofer R. Biostatistics, pp 229), and that these genes significantly vary due to culturing time without treatment. Genes symbols were described in materials and methods

To evaluate the effect of culture time on the stability of HKGs expression, candidate genes were quantified at 24 hours (24 hr) post culture relative to 4 hours (4 hr) in non-treated bovine BMCs. Expression of 6 out of 11 candidate genes in cultured BMCs, relative to the first time point, changed significantly (*P* ≤ 0.05) (Fig. 1). The expression of 18s, *β-2 microglobulin* (B2M), *Succinate dehydrogenase complex, subunit A* (SDHA), and *Tyrosine 3-monooxygenase/tryptophan 5-monooxygenase activation protein*, *zeta polypeptide* (YWHAZ) were significantly increased while expression of ACTB and *Glyceraldehyde 3-phosphate dehydrogenase* (GAPDH) were significantly decreased. Only the expression of 24 s, *C-terminal binding protein 1* (CTBP1), *Mouse double minute 4* (MDM4), PPIA, and *Radiation sensitive 50* (RAD50) were not statistically different than zero after 20 hours, showing constant expression during the culture time. Standard error (SE) of constantly expressed genes ranged from 0.42 up to 0.79 as 24s and RAD50 were the lowest and highest, respectively. Only the genes which were not affected by culturing time were selected for further evaluation in next step.

### The effect of *in-vitro* treatment and immunological status

To investigate the effect of *in-vitro* treatment and immunological status of the host on expression of candidate genes, BMCs from individuals with different immunological status (naïve, immunized, and boosted) were treated with the mitogen, ConA, and two structurally distinct antigens, HEWL, and *C. albicans*. Statistical results of each effect and the interaction of effects are summarized in Table 2.

**Table 2:** Probabilities of significance of fixed effects on the expression of candidate housekeeping genes in bovine blood mononuclear cells under different *in-vitro* treatment. Significant probabilities (*P* ≤ 0.05) are shown in bold

Candidate gene symbol*		**24s**	**CTBP1**	**MDM4**	**PPIA**	**RAD50**
**Source of variation**						
Mitogenic treatment (ConA)						
	Immunological Status^#^	0.2936	0.8759	0.5817	0.1220	0.9072
	Culture time^§^	0.2345	**0.0041**	**0.0213**	**0.0002**	**<.0001**
	Immunological Status*Culture time	0.4287	0.4041	**0.0036**	0.5697	**0.019**
Antigenic treatment						
Complex antigen (*C. albicans*)						
	Immunological Status^#^	0.3501	0.6967	**0.0032**	0.1602	0.0524
	Culture time^§^	**0.0420**	**0.0179**	0.3493	**0.0007**	**0.0009**
	Immunological Status *Culture time	0.4111	0.6621	**0.0161**	**0.0148**	0.8384
Single polypeptide (HEWL)						
	Immunological Status^#^	0.0578	0.3333	**0.0417**	0.1948	0.0415
	Culture time^§^	0.0970	0.1510	0.9049	**0.0055**	**0.6798**
	Immunological Status *Culture time	0.2852	0.2769	0.2026	0.8707	0.6076

*24s: Ribosomal protein S24; CTBP1: C-terminal binding protein 1; MDM4: mouse double minute 4 protein; PPIA: Peptidylprolyl isomerase A; RAD50: RAD50 homolog (S. cerevisiae). # General effect of immunological status includes naïve, immunized, and boosted groups. § General effect of culturing time included 4 and 24 hour time points. Bonferroni correction was not applied to this analysis since multiple comparison was not appropriate for all the fixed effect in this model

#### Altered expression of candidate genes due to mitogenic treatment is time dependent

**Fig. 2:**
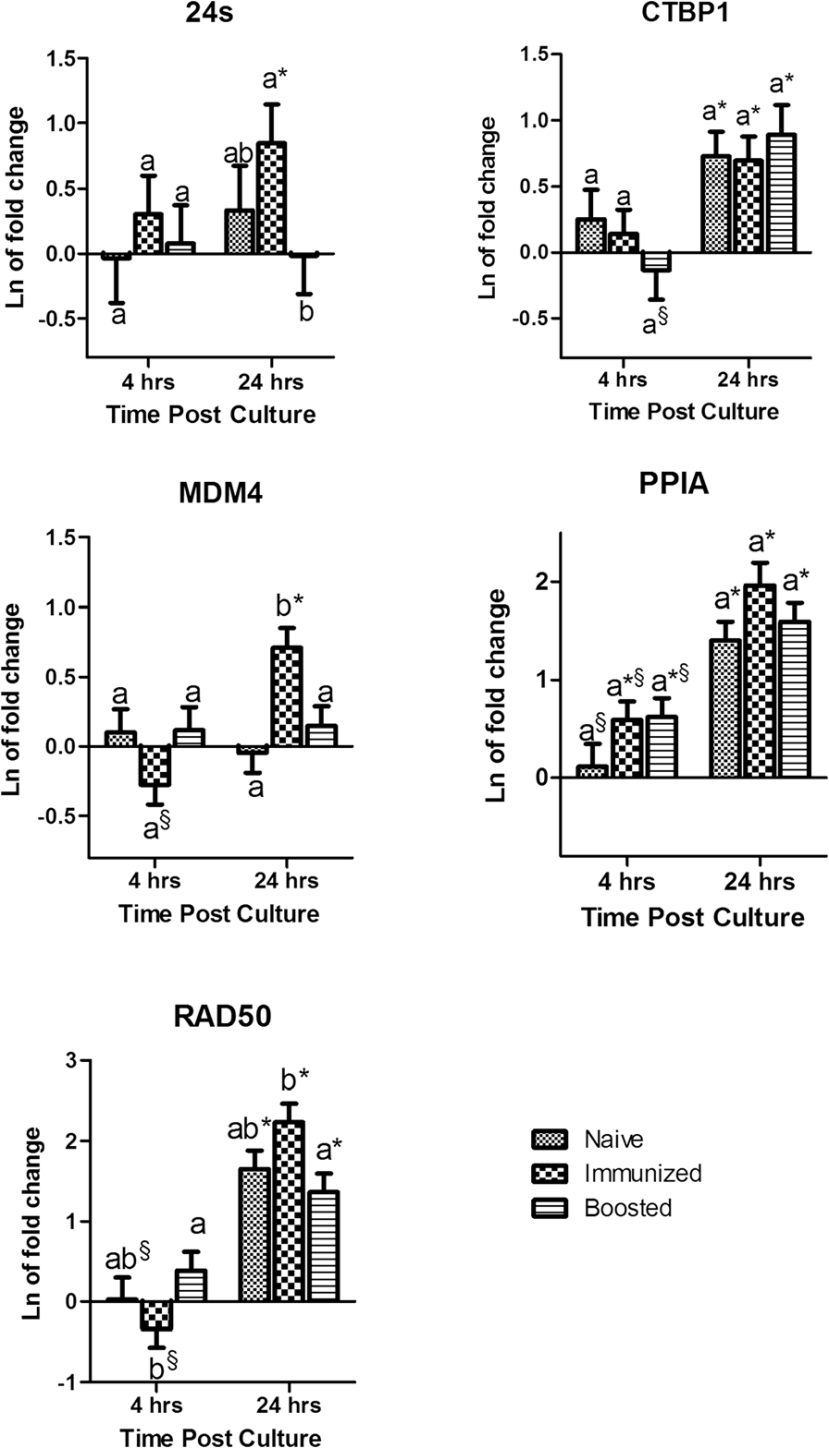
Concanavalin–A treatment: Mean and standard error of natural log of mRNA expression in blood mononuclear cells treated with Concanavalin–A (5 μg/mL) relative to untreated samples. ^a-b^ For each gene within a time point no common superscripts indicate significant difference between immunological status (P ≤ 0.05); § Represents significant differences within each immunological status between time points (P ≤ 0.05). * Indicates the mean is significantly (P ≤ 0.05) different from zero. Gene symbols are described in materials and methods

The overall fixed effect of immunological status was not statistically significant on the expression of 24s, CTBP1, MDM4, PPIA, and RAD50 when BMCs were stimulated with ConA (Table 2). On the other hand, the fixed effect of time was statistically significant for all genes, except 24s. The interaction between immunological status × culture time was significant for expression of MDM4 and RAD50 representing the effect of time for these two genes was depended on immunological status. Further statistical analysis to compare immunological status (e.g. naive, immunized, or boosted) at each time point revealed different expression of the candidate genes (Fig. 2). At the first time point (4 hrs), the expression of most genes was not significantly different between immunological status groups with the exception of RAD50. The expression of RAD50 was down regulated in immunized individuals, resulting in a significant difference from the boosted group. The expression of PPIA was not different between groups either at 4 hr or 24 hr. However, the cows with previous exposure to the test antigens (immunized and boosted groups) expressed PPIA significantly higher than zero (*P* ≤ 0.05) at the early time point (4 hr). At the second time point (24 hr), the expression of PPIA was significantly higher than zero (*P* ≤ 0.05) in all groups. Differences between immune response status groups at the second time point were not significant for CTBP1 and PPIA. On the other hand, the expression of 24s, MDM4, and RAD50 were significantly higher in the immunized group than boosted animals. Finally, when BMCs were cultured with ConA, MDM4 was the only gene which was expressed significantly different in the naïve group compared to the immunized group (Fig. 2).

#### Previous host exposure to test antigen is an additional determining factor in the stability of HKG expression following antigenic treatment

**Fig. 3:**
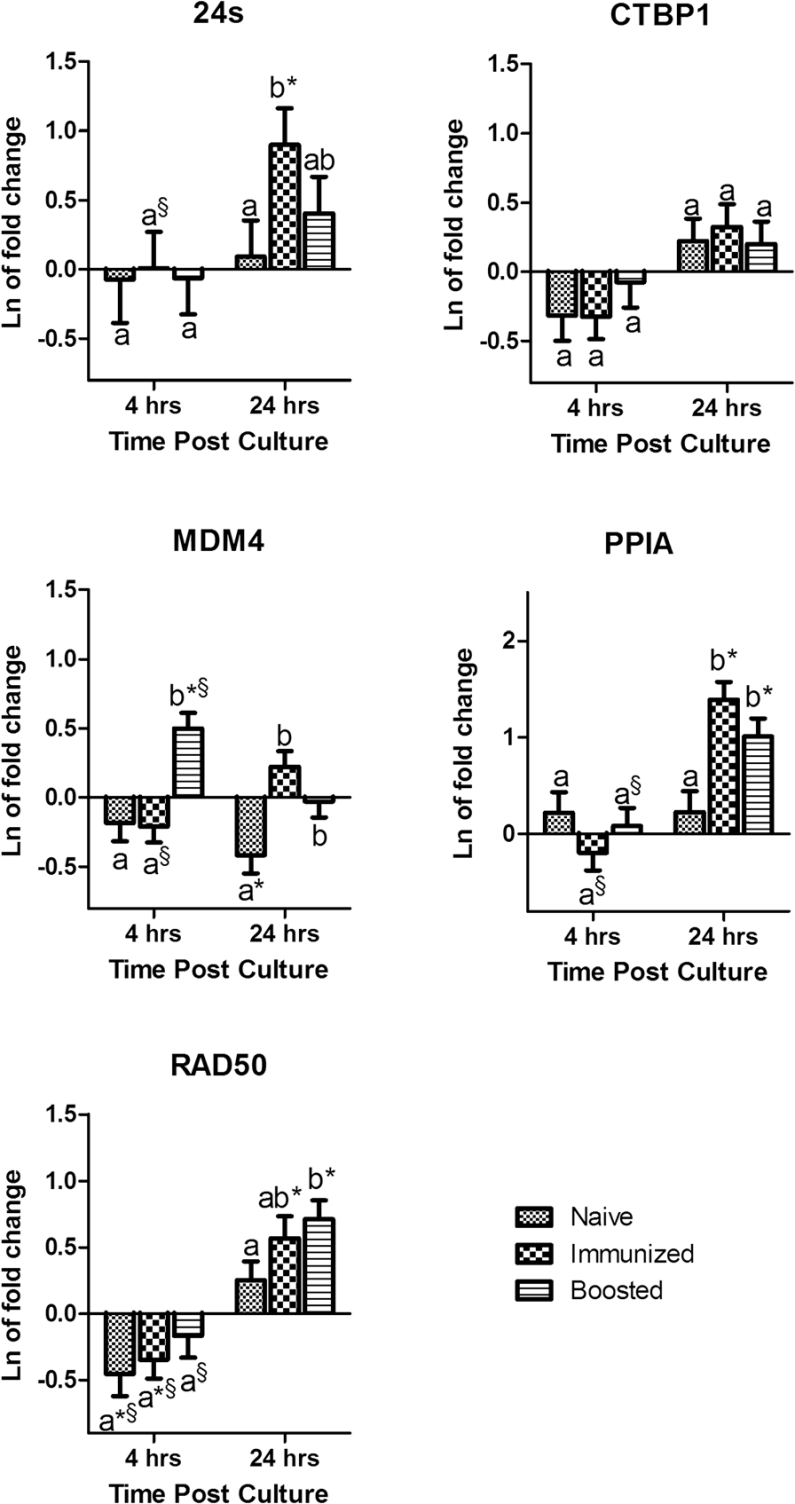
*Candida albicans* treatment: Mean and standard error of natural log of mRNA expression in blood mononuclear cells treated with freeze dried whole yeast extract of *Candida albicans* (20 μg/mL) relative to untreated samples. ^a-b^ For each gene within a time point no common superscripts indicate significant difference between immunological status (P ≤ 0.05); § Represents significant differences within each immunological status between time points (P ≤ 0.05). * Indicates the mean is significantly (P ≤ 0.05) different from zero. Gene symbols are described in materials and methods

The fixed effect of immunological status was significant (*P* = 0.003) for the expression of MDM4 when BMCs were stimulated with the *C. albicans* extract. This effect was a trend (*P ≤* 0.1) for the expression of RAD50. The fixed effect of culture time on the BMCs treated with *C. albicans* extract was significant for all candidate genes, except MDM4. The interaction effect of immunological status × culture time was significant for MDM4 and PPIA (Table 2). Significant differences in mRNA expression of candidate genes among samples with different immunological status was found, which was dependent on time (Fig. 3). The clearest effect of immunological status was observed on expression of PPIA at 24 hours after treatment with *C. albicans* extract. Both groups with previous exposure (immunized and boosted) had significantly higher expression than the naïve group and this expression was also significantly different than zero. This effect was also observed with a lower non-significant impact on all other candidate genes except CTBP1 (Fig. 3).

**Fig. 4:**
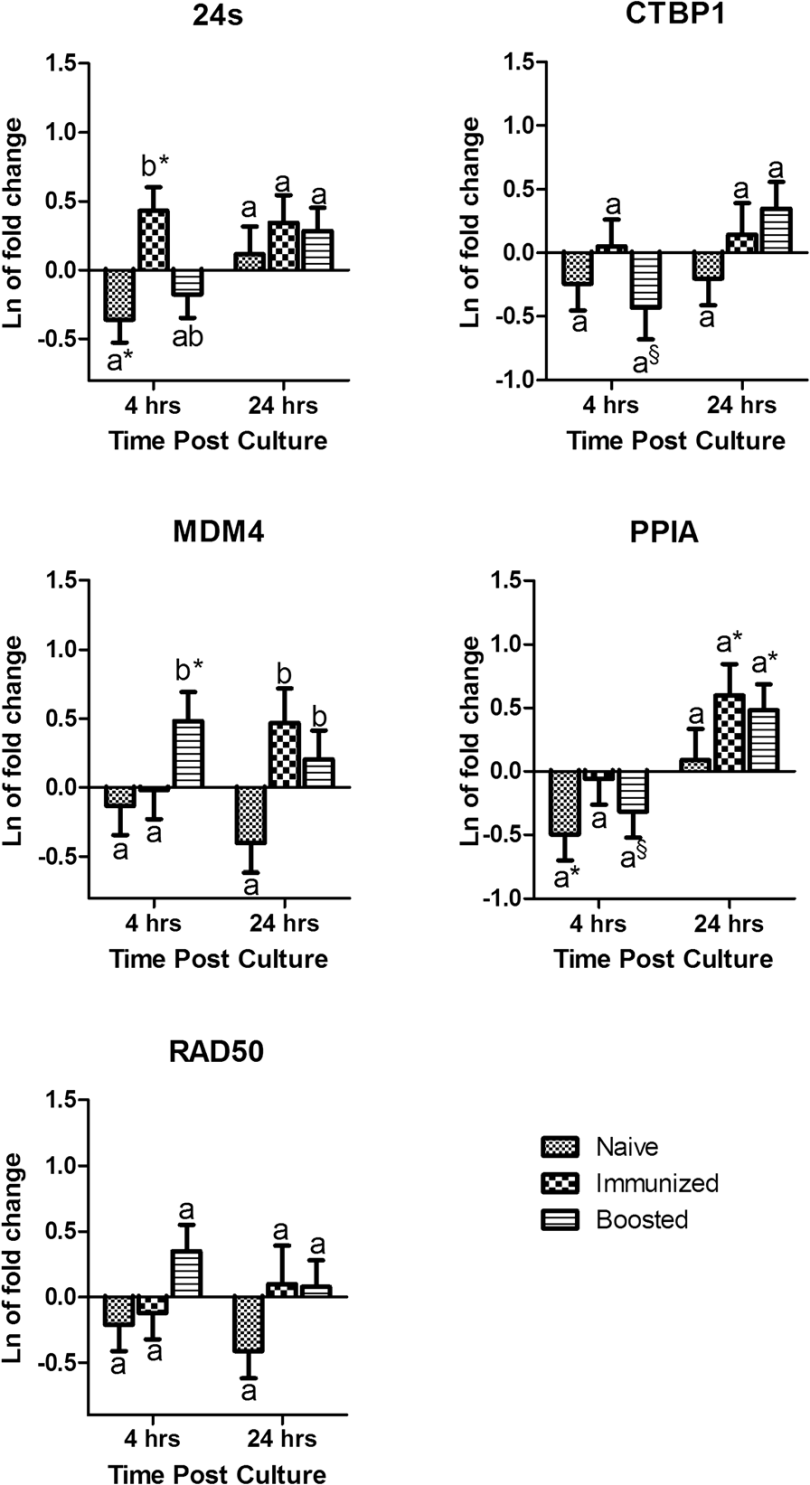
Hen egg white lysozyme treatment: Mean and standard error of natural log of mRNA expression in blood mononuclear cells treated with hen egg white lysozyme (20 μg/mL) relative to untreated samples. ^a-b^ For each gene within a time point no common superscripts indicate significant difference between immunological status (P ≤ 0.05); § Represents significant differences within each immunological status between time points (P ≤ 0.05). * Indicates the mean is significantly (P ≤ 0.05) different from zero. Gene symbols are described in materials and methods

Stimulation of BMCs with HEWL resulted in a similar mRNA expression profile (Fig. 4) to that of treatment by *C. albicans* extract (Fig. 3). The fixed effect of immunological status was significant for the expression of MDM4 and RAD50 (Table 2). In addition, immunological status tended (*P ≤* 0.1) to have an effect on the expression of 24 s. The effect of culture time was only significant for the expression of PPIA with a tendency for the expression of 24 s . The interaction of immunological status × culture time was not significant on the expression of any of the candidate genes (Table 2). However, the expression of MDM4 at 24 hours after treatment with HEWL was significantly higher in groups previously immunized with this test antigen (immunized and boosted groups) as compared to the naïve group. In addition, significant up regulation in the expression of PPIA was only observed in antigen exposed groups (immunized and boosted). No significant differences were observed between time points and immunological status on expression of CTBP1 and RAD50. The expression of 24 s was not different than zero at 24 hours after treatment with HEWL and it was not affected by immunological status (Fig. 4).

## Discussion

Normalization of real time RT-PCR data to a constitutively expressed gene is the ideal method to eliminate technical variations in relative quantification of mRNA. However, considerable variation has been recently reported in expression of housekeeping genes in different cells and tissue [3, 13, 14]. The expression of housekeeping genes in cultured primary cells is likely more prone to variation due to several steps of physical and/or chemical treatments to isolate the cells. Additionally, for the cells of adaptive immune system, immunological memory, the unique feature of adaptive immune system, can potentially affect the expression of housekeeping genes in these cells. The current study, for the first time, evaluated potential sources of inconsistency and variation in housekeeping gene expression including the effect of immunological memory in cultured bovine BMCs. Results of the study showed cell culture alone, and *in-vitro* treatment of the cells with a mitogen or test antigens significantly affects the expression of HKGs commonly used as reference genes. Notably, the effect of *in-vitro* antigenic stimulation of BMCs on some housekeeping genes depends on if and when the host has been previously exposed to the antigen.

Restricted maximum likelihood method in a linear mixed effects model was chosen to analyze the results in this study, despite of availability of numerous databases and algorithms such as Genevestigator, Bestkeeper, geNorm and NormFinder [15]. Bestkeeper and geNorm utilize similar pair-wise analysis without including any further classification of samples while NormFinder use a model based approach [16–18]. Regardless of similarity or differences between these programs, they all return the most stable gene(s) based on variation between samples or groups. However, the source of inconsistency remains undescribed in the results of these programs. The PROC MIXED procedure in SAS software, which was used in this study, provides the user options to include sources of variation in a randomized block or repeated measure design. In addition, post-hoc analysis revealed differences between the levels of each effect.

The expression of many housekeeping genes in different bovine cells or tissues, including cells [19], adipose tissue [9], pre-implantation embryos [10], cell lines [1] and primary cells of immune system have been extensively studied [6, 13]. Spalenza et al. have reported that YWHAZ, S24, and PPIA are the most stably expressed genes in bovine BMCs when the cells are freshly isolated and have not been cultured nor have undergone any *in-vitro* treatment [6]. In the current study, similar conditions resulted in similar finding, except for the expression of YWHAZ which was increased significantly after 24 hours culture. In another study by Goossens et al. YWHAZ along with SDHA and GAPDH were reported to be stably expressed in *in-vitro* fertilized bovine embryos up to seven days [10]. In contrast, none of these genes were stably expressed in cultured bovine BMCs, showing that the stability of HKGs expression depends on both the tissue and also the *in-vitro* environment. On the contrary, ACTB was found to be among the least consistently expressed genes in several studies, including the current study, despite the common use of this gene as a reference gene in relative expression studies [6, 9, 10]. These similarities and differences may be explained by cell adaptation to the *in-vitro* environment. Further, it is very likely that major physical and chemical differences between the *in-vivo* and *in-vitro* environment as well as cell injuries that may happen during the isolation procedure would alter the expression of genes that form the structure of cells, mediate cellular response to injury and or hyperoxia such as ACTB and SDHA [20–22].

Treatment of either cells or the host, whether *in-vitro,* or *in-vivo*, is another potential source of variation in the expression of housekeeping genes. Ostrowska et al. demonstrated that oral glucogenic feed supplementation caused variation in housekeeping gene expression in bovine liver. Notably, ACTB was the least stably expressed gene in their study [23]. In alignment with these results, but in an *in-vitro* study, it has been shown that normalization against different reference genes will significantly affect the result of IL10 and TNF-α expression in lipopolysaccharide treated monocytes [13]. Likewise in the current study, significant changes were observed in gene expression after *in-vitro* treatment, which could potentially change the results of quantification if they were used for normalization. Only the expression of CTBP1 remained unchanged after *in-vitro* treatment of BMCs with Con A, *C. albicans* extract, or HEWL (Figs. 2, 3, and 4). In addition, previous exposure of the host to the antigen used for stimulation and culturing time significantly affected the expression of tested housekeeping genes (Table 2). A notable contrast was observed on the effect of the host by comparing mitogenic versus antigenic stimulation. The immunological status of the host did not have a significant effect when BMCs were treated with the mitogen Con A, however immunological status was significant on the expression of some HKGs under antigenic treatment (Table 2). Con A is a mitogen that nonspecifically stimulates T lymphocytes whereas antigens stimulate specific lymphocyte clones via T cell receptors (TCR) [24]. Thereby, as expected immunological memory affects responses of lymphocyte to antigenic stimulation but not mitogen. Furthermore, the effect of the host became greater the longer the cells were in culture. For instance, the expression of PPIA under treatment with both test antigens were significantly higher than zero only in groups that had been previously exposed to the antigens. The same was the case for RAD50 and 24 s under treatment with *C. albicans* (Figs. 3 and 4). In contrast, no significant difference was observed in expression of the tested housekeeping genes between individuals recently exposed to the test antigens or individuals exposed 2 years before sampling, with the exception of MDM4 (Figs. 3 and 4). MDM4 expression was significantly increased 4 hours post antigenic treatment in BMCs when individuals had been boosted one week prior to sampling, compared to untreated control. This increase is likely due to the anti-apoptotic role of this gene in lymphocyte proliferation in response to antigens [25, 26].

The stability of reference gene(s) expression over time after *in-vitro* treatment is critical when quantification of expression is relative to time zero or use of the kinetics of expression are intended. Both designs are common in gene expression studies but the stability of expression in these designs are often overlooked. Anstaett et al. have shown that B2M and YWHAZ expression are most stable among 8 candidate genes using Bestkeeper and geNorm programs in BL3 cells up to 48 hours post infection with Bovine Viral Diarrhea Virus. But, the expression of ACTB, and hypoxanthine-guanine phosphoribosyltransferase (HPRT) were reported from the same study to be the most stable when NormFinder was used [1]. This inconsistency may be due to different approaches and lack of compatibility of these software with multi-dimensional experimental designs. However, the results of the current study from statistical modeling showed that CTBP1 is the only constantly expressed gene after treatment of BMCs with *C. albicans* and CTBP1 and RAD50 are constantly expressed after stimulation of BMCs with HEWL up to 24 hours post treatment (Figs. 3 and 4). In another example, the expression of PPIA, which was shown to be constant by Spalenza et al. as well as in the first part of the current study, was significantly up regulated at 24 hours post *in-vitro* treatment. Con A stimulation during culture time also caused significant up regulation of all tested genes but varied among different host group for 24 s and MDM4 (Fig. 2).

Overall, it was determined from this study that 24 s under ConA stimulation, CTBP1 under *C. albicans* treatment, and RAD50 under HEWL treatment are reliable genes to be used as reference genes in bovine BMCs. Although other studies have suggested to use a combination of housekeeping genes, the proposed genes recommended for use here, appear stable under the studied condition to be used alone as reference genes. If expression of a gene under different treatments is intended, using the geometric average of two or more housekeeping genes that are adversely affected by the experiment may provide a suitable normalizer. However, under the current experimental design and for comparison within a treatment, using one gene will provide accurate quantification. The results of this study have also indicated that suitable HKGs should be screened when BMCs from outbred species are treated with antigens prior to gene quantification by real time qPCR. This is especially important in case – control studies, when individuals used as controls may not have encountered the same antigens that case individuals have been exposed to.

## Conclusion

The current study for the first time evaluated the effect of previous *in-vivo* antigenic exposure of the host on expression of HKGs in cultured BMCs. In agreement with previously published papers, this study did not find any common HKGs expressed constitutively under all *in-vitro* treatments, as well as over time in bovine BMCs. However, 24 s, CTBP1, and RAD50 were constantly expressed independent from previous exposure of the host under *in-vitro* treatment with ConA, *C. albicans,* and HEWL, respectively. The current study revealed that the immunological status of the host can be an important potential source of inconsistency when primary cells of the adaptive immune system are studied in samples from un-controlled environments common to outbred species. This is particularly true when cells are also exposed to antigenic *in- vitro* treatment. The combination of previous environmental exposure, *in-vitro* treatment, and culturing time are sources of variation that can alter the expression of housekeeping genes which if used for the normalization of gene expression data can inadvertently generate misleading results.

## Materials and Methods

### Animal and Immunization Protocol

A total of 8 cows from the University of Guelph research station were enrolled in the study and divided into 2 groups: test and control. The selection criteria aimed to minimize variation due to environmental effects by selecting animals with the same age, parity number, and stage of lactation. Cows in the test group (n = 4) were immunized two years before the first sampling (Immunized group) with 0.5 mg hen egg white lysozyme (HEWL) (Sigma Aldrich, USA), 0.5 mg *Candida albicans* extract (Greer Laboratories, USA), and 0.5 mg Quil A adjuvant (Cedarlane Laboratories, Canada) suspended in 1 ml PBS (pH 7.4). The test group received a booster shot with the same antigens one week before the second sampling (Boosted group). Control samples (Naïve group) were collected from 4 individuals with the same physiological background, including age, stage of lactation, and parity number, as the test group. Although, individuals in the control group were not immunized with HEWL nor *C. albicans*. The Animal Care Committee of the University of Guelph approved all experimental procedures under guidelines of the Canadian Council of Animal Care.

### Blood mononuclear cell isolation, culture, and treatment

Blood mononuclear cells (BMCs) were isolated by gradient centrifuge method from whole blood samples. Briefly, the same volume of blood was overlaid onto Histopaque 1077 (Sigma-Aldrich, St. Louis, MO), and centrifuged at room temperature for 30 min at 400 × g. The layer of cells over Histopaque was collected and washed two times with PBS 1×. The isolated BMCs were re-suspended at the concentration of 2.5 × 10^6^ cell/mL in phenol red free RPMI1640 (Sigma-Aldrich, St. Louis, MO) supplemented with 10 % fetal bovine serum (HyClone, Logan, UT), and 1× Penicillin-Streptomycin Solution (HyClone, Logan, UT). BMCs were cultured in 48-well plates and each sample was treated with HEWL (Sigma-Aldrich, St. Louis, MO), or freeze dried whole yeast extract of *Candida albicans* (Greer Laboratories, USA) at the final concentration 20 μg/mL or with Concanavalin–A (ConA) at the final concentration of 5 μg/mL (Sigma-Aldrich, St. Louis, MO), for a total of 3 different treatments. In addition, one untreated sample was included as a control for each time point. Cells were harvested at 4 and 24 hours post incubation and stored in TRIzol (Ambion, Carlsbad, CA) at −80 °C.

### RNA extraction and reverse transcription

Total RNA was extracted from each sample individually which were previously stored in TRIzol according to the manufacturer’s protocol. Residual DNA was removed by treating samples with TURBO DNA-*free*® (Ambion, Carlsbad, CA) kit. Then, 500 ng of RNA was used for cDNA synthesis using Superscript® III First Strand Synthesis kit (Invitrogen, Burlington, ON), and random hexamer primers, according to the manufacturer’s protocol.

### Primer design and Real-time RT-PCR

RNA expression stability of 9 candidate genes from various biological pathways, which are commonly used as reference genes in gene expression studies, were tested in this study. In addition, 2 candidate genes, MDM4, and RAD50, which have been reported as housekeeper genes in lymphocyte T-helper 1 and 2 cells were evaluated [27, 28]. The list of genes and their corresponding biological pathways are summarized in Table 3. Primers were designed using Primer3web software (Ver. 4.0). The sequences of the primers are listed in Table 3. Quantitative real-time PCR was performed on diluted (1:10) cDNA from each individual using 2× DNA Master SYBR Green I and 250 nM of each primer in 384-well plate in LightCycler® 480 II machine (Roche Diagnostics, USA). To minimize run-to-run variation, each plate was used to test up to three genes per run depending on primers annealing temperature. All samples (n = 96 for each gene) including all treatments (three treatment and one control groups), two time points, and individuals from different immunological statuses (n = 12, four sample/group) were run in one plate. In addition, the same batch of master mix was used for all the amplifications. The amplification conditions consisted of pre-incubation for 10 min at 94 °C, followed by 45 cycles of 95 °C for 10 s, 54–60 °C (based on annealing temperature of each set) for 10 s and extension and signal acquisition at 72 °C for 10 s. Melting curve analysis was done in three steps; 95 °C for 10 s, cooling to 65 °C for 1 min and heating to 97 °C. To remove inter assay variation all the treated and control samples, animal groups, and both time points for each gene were quantified on one plate. To determine the efficiency of amplification for each gene, a dilution series of cDNA pool was tested with the same procedure as mentioned above.

**Table 3:** Candidate genes characteristics and primers sequence

**Symbol**	**Gene name**	**Function**	**Primer sequence**	**Ref**
**18s**	18S ribosomal RNA	Ribosome unit	F: 5'-AGA AAC GGC TAC CAC ATC -3'	Primer3web
			R: 5'-GGA CTC ATT CCA ATT ACA GG -3'	
**24s**	Ribosomal protein S24	Ribosome unit	F: 5'-TGT CAT CTT TGT ATT TGG GTT CAG -3'	Primer3web
			R: 5'-TCT GTT CTT GCG TTC CTT CC -3'	
**ACTB**	Actin, beta	Structural protein	F: 5'- GCT TCT AGG CGG ACT GTT AG -3'	Primer3web
			R: 5'-ACT TGG GAA TGC TCG ATC C -3'	
**Β2M**	Beta-2-microglobulin	Beta chain of MHC I	F: 5'-TTA CCT GAA CTG CTA TGT GTA TG -3'	Primer3web
			R: 5'-CTG TAC TGA TCC TTG CTG TTG -3'	
**CTBP1**	C-terminal binding protein 1	Involved in cellular proliferation	F: 5'-ACA ACC ACCACC TCA TCA AC -3'	Primer3web
			R: 5'-AGC CTT CTC GTC CAC CAG -3'	
**GAPDH**	Glyceraldehydes-3-phosphate dehydrogenase	Glycolysis and gluconeogenesis	F: 5'-GTT CAA CGG CAC AGT CAA G -3'	Primer3web
			R: 5'-TAC TCA GCA CCA GCA TCA C -3'	
**MDM4**	mouse double minute 4 protein	p53 binding protein, Housekeeping gene in Th1 cells	F: 5'-GGA GAA CTA CTA GGT CGT CAG AG -3'	Primer3web
			R: 5'-CCT GTG CGA TAG CGA GAG TC -3'	
**PPIA**	Peptidylprolyl isomerase A	Protein folding	F: 5'-TGA CTT CAC ACG CCA TAA TGG T -3'	Bevilacqua C. et al. 2006 [30]
			R: 5'-CAT CAT CAA ATT TCT CGC CAT AGA -3'	
**RAD50**	RAD50 homolog (*S. cerevisiae*)	DNA double-strand break repair, Housekeeping gene in Th2 cells	F: 5'-CAC AAT CAC CAG AGA ACA GTA AGG -3'	Primer3web
			R: 5'-CTG ATG ACG ATC TGC TTG TAG TTG -3'	
**SDHA**	Succinate dehydrogenase complex, subunit A	Electron transporter	F: 5'-CGT TGT ATG GAA GGT CTC TG -3'	Primer3web
			R: 5'-GTG GCG ATG ACA GTG TTC -3'	
**YWHAZ**	Tyrosine 3-monooxygenase/tryptophan 5-monoxygenase activation protein, zeta polypeptide	Signal transduction by binding to phosphoserine-containing proteins	F: 5'-GAC TAC TAC CGCTAC TTG GCT GAG -3'	Primer3web
			R: 5'-GCT TCT TGG TAT GCT TGC TGT GAC -3'	

### Statistical analysis

Statistical analysis of the amplification performance was carried out using SASqPCR macro code in SAS STAT software (Ver. 9.3) [12]. The relative expression of each gene was calculated based on equation published by Pfaffl in 2001 [29]. Briefly, the first time point was considered as the control sample for testing the expression over time in untreated samples. The ratios from this calculation were log transformed and statistically analyzed by two-tailed Student’s-t distribution to show if the average expression for each gene was statistically different than 0.

To investigate the effects of immunological status, culture time, and their interaction on the RNA expression stability, untreated samples at each time point were assigned as the control in Pfaffl’s equation. Subsequently, ratios from Pfaffl’s equation were normalized based on starting concentration of total RNA and log transformed. Data for each gene were analyzed independently using the PROC MIXED procedure in SAS and included repeated measures. Least square means from the model were used in Tukey’s HSD Post-hoc test to evaluate the difference between the levels of each effect (treatment, time etc..).

The statistical model was as follows:

$$ {y}_{ij} = \mu + {h}_i + {t}_j + {d}_{ij} + {(ht)}_{ij} + {e}_{ij} $$

Where *y*_*ij*_ is the vector of ln-transformed fold change of mRNA expression in *i*^th^ immunological status at *j*^th^ time point; μ is the overall average of ln-transformed fold change of mRNA expression; *h* is the fixed effect of immunological status, *t* is the fixed effect of time point; *d*_*ij*_ is the random effect of animal; *(ht)*is the interaction effect; *e*_*ij*_ is the vector of error. Bonferroni correction was not applied to this analysis since multiple comparison was not appropriate for all the fixed effect in this model. However, Tukey’s test was used to determine significance of each level of fixed effects.

## Abbreviation

18s: 18S ribosomal RNA
24 s: Ribosomal protein S24
ACTB: Actin, beta
Β2M: Beta-2-microglobulin
BMCs: Blood mononuclear cells
ConA: Concanavalin–A
Cq: Quantification cycles
CTBP1: C-terminal binding protein 1
GAPDH: Glyceraldehydes-3-phosphate dehydrogenase
HEWL: Hen egg white lysozyme
HKGs: Housekeeping genes
MDM4: mouse double minute 4 protein
PPIA: Peptidylprolyl isomerase A
RAD50: RAD50 homolog (S. cerevisiae)
SDHA: Succinate dehydrogenase complex, subunit A
StdErr: Standard error
YWHAZ: Tyrosine 3-monooxygenase/tryptophan 5-monoxygenase activation protein, zeta polypeptide
